# Transcutaneous Vagal Stimulation in Knee Osteoarthritis (TRAVKO): Protocol of a Superiority, Outcome Assessor- and Participant-Blind, Randomised Controlled Trial

**DOI:** 10.3390/ijerph20010311

**Published:** 2022-12-25

**Authors:** Claudio Bascour-Sandoval, Rubén Gajardo-Burgos, Claudio Muñoz-Poblete, Pablo Riedemann-González, Stephanie Erices-Salas, Agustín Martínez-Molina, Germán Gálvez-García

**Affiliations:** 1 Departamento de Ciencias de la Rehabilitación, Universidad de La Frontera, Temuco 4780000, Chile; 2 Programa de Magister en Terapía Física Mención Musculoesquelética, Departamento de Ciencias de la Rehabilitación, Universidad de La Frontera, Temuco 4780000, Chile; 3 Instituto de Aparato Locomotor y Rehabilitación, Facultad de Medicina, Universidad Austral de Chile, Valdivia 5090000, Chile; 4 Departamento de Medicina Interna, Universidad de La Frontera, Temuco 4780000, Chile; 5 Consultorio Miraflores, Temuco 4790706, Chile; 6 Departamento de Psicología Social y Metodología, Facultad de Psicología, Universidad Autónoma de Madrid, 28049 Madrid, Spain; 7 Departamento de Psicología, Universidad de La Frontera, Temuco 4780000, Chile; 8 Departamento de Psicología Básica, Psicobiología y Metodología de las Ciencias del Comportamiento, Universidad de Salamanca, 37008 Salamanca, Spain

**Keywords:** osteoarthritis, knee, pain, pain management, transcutaneous vagal stimulation, exercise, clinical trial, randomized controlled trial

## Abstract

Current treatments for knee osteoarthritis (KOA) are partially effective. It is, therefore, necessary to find new strategies that can complement the existing ones. In this scenario, transcutaneous vagal stimulation (TVS) neurophysiological effects could be a helpful solution. However, there is no evidence of the efficacy of TVS in KOA. This trial aims to assess the efficacy of TVS in decreasing pain in participants aged 55 years or older with KOA. A randomised controlled, two-arm, double-blind (participants and outcome assessors) and clinical superiority trial will be conducted for 70 patients with KOA. All the participants will carry out an exercise program. It consists of 12 sessions over four weeks. In addition, they will be randomly assigned to (1) active TVS plus physical exercise or (2) sham TVS plus physical exercise. The application of active TVS consists of electronic stimulation of the auricular concha using a portable device. Sham TVS condition consists of the stimulation of the earlobe that does not cause neurophysiological effects. The primary outcome is the reduction in pain intensity. Additionally, functional capacity, physical performance, pain-related interference, pain-related distress, quality of life in older adults and global change will be measured. Assessments will be conducted at the beginning of the study (baseline), at the end of the intervention and after 1 and 3 months of follow-up. This trial will generate evidence regarding the efficacy of TVS in pain perception in individuals with KOA. This information will serve as an input in the clinical decision-making on the use or non-use of TVS in individuals with KOA. Thus, if the efficacy of TVS is confirmed, a new therapeutic tool may be included in the rehabilitation of individuals with KOA.

## 1. Introduction

Osteoarthritis is the most common chronic joint disorder. In this regard, osteoarthritis is the fourth leading cause of disability worldwide in 2020 and the most common joint disease, with knee Osteoarthritis (KOA) being the most common, representing 83% of the total OA burden [1], with most cases presenting chronic pain (i.e., more than 3 months). Thus, several studies report that 90% of the population has had knee pain for more than a year [2]. Thus, KOA is one of the main musculoskeletal health problems in the population [3], affecting 16% in individuals aged 15 and over and was 22.9% in individuals aged 40; the incidence is 203 per 10,000 person years; and the ratios of prevalence and incidence in females and males were 1.69 and 1.39, respectively [4]. KOA entails high health-related costs with subsequent economic expenditures and loss of work productivity [5,6].

For the treatment of KOA, pharmacological treatment had shown limited effects [7,8]. Regarding non-pharmacological treatments, there is no evidence of the benefit of arthroscopic surgery [9,10]. Moreover, physiotherapy-based rehabilitation has shown only partially successful results [11].

A meta-analysis showed that therapeutic exercise programs on land (i.e., not performed in an aquatic environment) provided a short-term benefit for knee pain and functional capacity [12]. However, although rehabilitation is widely accepted as beneficial for KOA, it has demonstrated a small to moderate effect size, similar to standard drug treatment. Consequently, both therapeutic alternatives (i.e., exercise and drugs) show partially effective results with a reduction in pain. Thus, the pain persists, affecting’ patients’ quality of life and work ability.

It is important to note that the mechanisms of chronic pain present in subjects with KOA differ from those with acute pain. Thus, the same nociceptive stimulus may individually cause more pain perception in chronic pain than acute pain. This would be associated with changes in the central nervous system (CNS), which is manifested by a lower intracortical capacity for pain inhibition due to a decrease in the activation of the endogenous system in the modulation of pain [13] regardless of the structural damage caused by the pathology [14]. In this scenario, new techniques have emerged acting on the CNS, such as transcutaneous vagal stimulation (TVS), which has shown an effect in reducing pain [15,16].

TVS belongs to a group of techniques based on neuromodulation. Neuromodulation is a physiological process that consists of an alteration of the neural and synaptic properties of the neurons or substances released by them [17]. Neurostimulation through TVS impacts on the release of neurotransmitters, and thus on the activity of the CNS. In recent years, low-cost, portable, easy-to-use equipment has been developed, which has made it possible to study vagal stimulation in several settings. This technique consists of ‘mild electrical stimulation of the ear’s external surface regions that are innervated by the auricular branch of the vagus nerve.

Pain modulation by TVS has been examined in animals and humans [18,19]. TVS has an impact on the central pain processing centres rather than just peripheral nociceptor activity [18]. Consequently, thick-myelinated fibres of vagus nerve branches that project to the nucleus tractus solitarii and the locus coeruleus in the brainstem are excited. The latter would reach structures related to spinal [20] and supraspinal [21] mechanisms that are involved in the modulation of arousal and pain perception [22]. That descending vagal signals activate anti-inflammatory pathways that suppress pro-inflammatory’ cytokines’ secretion (e.g., TNFα and IL-1β) [23]. In this regard, it has been described the “anti-inflammatory cholinergic pathway” in which efferent vagal fibres are activated by afferent ones (see more information in Lauwers et al., [24]). This becomes relevant in light of findings describing the role of inflammation in Osteoarthritis [25]. On the other hand, studies of TVS in healthy participants [15] with pelvic pain [26] or with chronic headaches [27] have confirmed its effect on pain modulation.

As a critical issue, it should be noted that there is only one study focused on the effectiveness of TVS in osteoarthritis [16]. In this pilot study, the impact of TVS on Erosive Hand Osteoarthritis was studied. The authors found that TVS decreased pain and increased motor function with a reduction of the number of tenderness joints and lower scores in the functional Index for Hand [28].

Thus, this study aims to address this gap in the literature. Specifically, we will study whether TVS could decrease pain and increase functional capacity in a corroborated KOA psychical exercise programme for the knee osteoarthritis (KOA) population. It is important to emphasise that we will focus on KOA as the joint most affected by OA is the knee, which affects 21.7% of women and 11.9% of men over 60 [4].

### Objective

As previously noted, to assess the efficacy of TVS in decreasing pain in participants aged 55 years or older with KOA.

## 2. Materials and Methods

### 2.1. Ethical Considerations and Trial Registration

This project was approved by the Scientific Ethics Committees of the Universidad de La Frontera (ACTA N°120_19) and countersigned by Southern Araucanía Health Service (Folio Numbering-00000040). It was carried out in compliance with the principles of the Declaration of Helsinki [29]. All participants must sign an informed consent form. The data will be registered with code (i.e., participant’s ID) to maintain the participant’s confidentiality. All records with names or other personal information that may identify participants will be kept separately from records with the participant ID. Access to this data will be limited to principal investigators. All records (registration forms and questionnaires) will be stored in a box with restricted access to the research team. All digital files will be kept in a secure computer accessible only to the research team with a password. A complete data backup will be done monthly. After 5 years, the destruction and/or erasure of data shall be carried out.

This study is registered at https://clinicaltrials.gov/ (accessed on 18 October 2022, Trial registration: NCT04381624).

### 2.2. Design

A double-blind, randomised controlled clinical trial will be conducted (See Table 1). The accessible population will consist of participants attending three public health centres in Temuco, Chile. The health centres were selected according to the following criteria: (a) the physical proximity of the centre, (b) they meet regulated standards of practice (i.e., they provide a similar type of social support and health care), and (c) they are the place where our study population receives care, and a sufficient number of patients for our study is ensured. Consolidated Standards of Reporting Trials (CONSORT) [30], The Standard Protocol Items: Recommendations for Interventional Trials reporting guidelines (SPIRIT) [31] and OARSI clinical trials recommendations [32] were implemented in the writing of this protocol.

### 2.3. Assessments

Assessments will be conducted at the beginning of the study (baseline), at the end of the intervention and after 1 and 3 months of follow-up.

### 2.4. Study Population, Recruitment and Assignment

The target population is patients aged 55 and over with a medical diagnosis of KOA [33,34] and chronic pain (i.e., three months or more of knee pain) from primary health centers in Temuco (Chile).

Health care professionals at the health centres will recruit eligible patients during visits or medical care consecutively. They will be referred to health centre staff, who will inform them of the ‘study’s purposes, requesting some means of contact for the research team to communicate with them. Moreover, they will be briefed on the investigation aims and asked if they wish to participate. If the individual agrees to participate, they will meet with the researchers, who will verify enrolment eligibility criteria. When the individual agrees to participate, an initial evaluation of sociodemographic and clinical characteristics will be made. The recruitment period will be extended until the determined sample size is met. One of the investigators will be responsible for continually visiting (i.e., once a month) the health centres in order to monitor the recruitment process.

### 2.5. Groups & Randomisation

Patients will be divided into two groups: an experimental group (active TVS plus physical exercise) and a control group (sham TVS plus physical exercise).

The allocation to the experimental or control group (EG and CG, respectively) will be carried out before starting treatment. It will be made by an external researcher who is not involved in any phase of the study, and only he/she will have full access to this information. Permuted blocked randomisation with a 1:1 randomisation ratio will be performed using Stata v16. Allocation will be concealed using a numbered opaque envelope.

### 2.6. Masking

**Figure 1:**
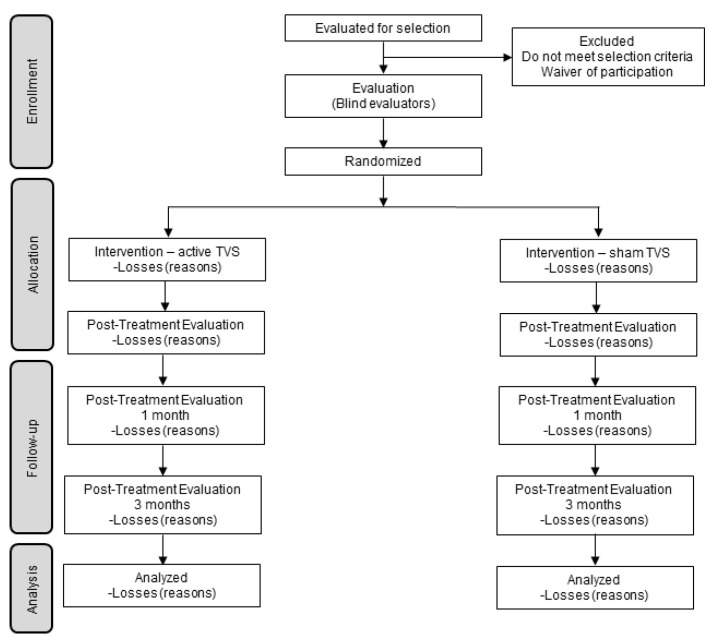
Progress flowchart.

Participants will be masked as to the study hypotheses, as they will not be informed about the group to which they were assigned. Likewise, the evaluators (who assess at the end of therapy and the follow-up at 1 and 3 months) will not know which group the participants were assigned. Participants and evaluators performing the various assessments will be asked about the group they believe participants were assigned. The masking of evaluators and patients will be maintained until the end of the investigation and data processing. See Figure 1.

### 2.7. Sample Size

Sample size was calculated using G*Power 3 [35], a computer application for computing statistical power analysis. One of the key aspects to determine the sample size was the magnitude of the standardized average effect. According to Tubach et al. [36] the reductions on the VAS responses (the main outcome variable) were large (SD of change = 21.5 mm). Based on this result, we expect a large effect size (0.4 according to Cohen’s *f* criteria; at least 8.6 mm improvement in the VAS). It means a considerable reduction on the VAS responses because the co-intervention (the exercise). Within the F test family, an ANCOVA was selected as a priori statistical test for which the sample size was estimated (two groups, one covariate, Cohen’s effect size *f* = 0.40, α = 0.05, 1 − *β* = 0.80). Based on these assumptions, a total of 64 participants were needed to detect the hypothesized difference between groups. We also expected a 10% dropout rate, so 6 participants were added to the computed sample size (a total of 70 patients; 35 in each group).

### 2.8. Selection Criteria

See Table 2 for detailed information.

## 3. Results

### 3.1. Descriptive Data

The following data will be collected: age, sex, BMI, duration of pain in months, previous treatments, current symptoms in other joints, comorbidities, use of medications, treatment expectations, educational level, individual and per capita income, level of education attained by the main breadwinner and occupational category of the main breadwinner.

### 3.2. Primary Outcome

The intensity of the pain will be evaluated through the VAS [37]. The average of the last seven days (VAS-7D) will be asked to rate their pain by marking on a continuous line of 100 millimetres (0 = “no pain” and 100 = “worst imaginable pain”).

### 3.3. Secondary Outcomes

Moreover, the intensity of the pain at rest (VAS-R) and walking on flat ground (VAS-W) will be evaluated. Functional capacity will be assessed through the Western Ontario and McMaster Universities Osteoarthritis Index (WOMAC). In addition, physical performance will be measured (gait speed test [38], 30-s chair stand test, timed up and go test and unipodal stance task), pain-related interference (VAS [39]), pain-related distress (VAS [39]), pressure pain threshold and quality of life in older adults (WHOQOL-BREF), global change (Patient-perceived satisfactory improvement; PPSI), global patient assessment (5 points Likert scale), and session attendance (physical therapist treatment notes). Finally, adverse events will be recorded (i.e., perception of any problem that the participants attribute to their participation in the study, which causes absence from two or more sessions).

### 3.4. Other Measurements

A variety of other measures will be collected further to analyse possible moderators and mediators of clinical effects. These measures will not be used to determine treatment efficacy. These measures include kinesiophobia (Kinesiophobia TAMPA Scale), sleep quality (Pittsburgh Sleep Quality Index), pain catastrophism (Pain Catastrophism Scale [40]), negative affectivity (DASS-21), expectation for overall improvement (5-point Likert scales), and self-efficacy (Chronic Pain Self-Efficacy Scale). Finally, the medication during treatment sessions should be reported.

### 3.5. Procedures

#### 3.5.1. Evaluations

Two physiotherapists blinded to the intervention (i.e., other than the treating physiotherapists) will perform the initial assessments, assessments at the end of the intervention and assessments at 1 and 3 months of follow-up. The research team will train them in a 4 h training for the application of the evaluation. The evaluations will be carried out in the Physiotherapy Laboratory at the Universidad de La Frontera. The results of the evaluators’ measurements will be recorded on a paper registration form specially designed for the study. This registration form will have a numerical code for each participant to avoid their identification. For the self-report questionnaires, the evaluators will check the data submitted by the participants to verify the correct and complete data entry.

For the evaluation, only the affected knee will be focused on. For participants with bilateral symptoms, the single knee will be the one with the most symptoms. In the case of presenting similar symptoms in both knees, the right one will be considered dominant.

#### 3.5.2. Interventions

Two physiotherapists will provide the intervention. They will be trained in a 6 h training before the start of the study. ’They will also be given written material with the intervention’s details (exercises, progression, etc.). The researchers will provide the necessary documents for the execution of the study. New physiotherapists will follow the same training and monitoring procedure if required. Participants will continue with the treatment indicated by the treating physiotherapist, being registered by the study staff.

On the other hand, they will be instructed to avoid therapies complementary to the one indicated by the doctor (i.e., balneotherapy, reiki, chiropractic) and inform the treating physiotherapist if they do so.

**Figure 2:**
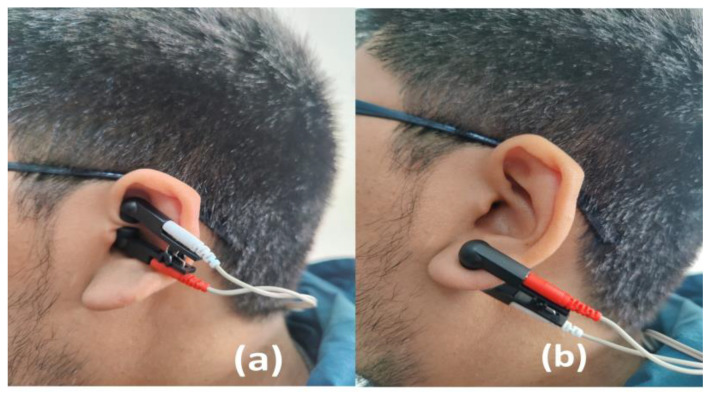
Active (**a**) and Sham (**b**) conditions of TVS.

*Transcutaneous Vagal Stimulation*: TVS will be applied, in line with other research [15,16,18,41], through a portable TensMed S82 electrostimulation device (Enraf Nonius). The auricular electrode clips (Auricular Clips, Body Clock Health Care Ltd., London, UK) will be placed for active condition in the left ear (see Figure 2)., specifically in the auricular cymba concha, given that stimulation of the left vagus nerve has not been shown to cause abnormal activity in the heart [42]. That stimulation of the right vagus nerve could affect the heart’s activity as it has direct efferent fibres with projection to this organ [43]. The device will be programmed with a biphasic square current at an intensity that produces a clear tingling sensation that is neither uncomfortable nor painful [41], with pulses of 300 microseconds, at 25 Hertz, the stimulus will be present for 30 s and will be followed by a 30-s rest period [15,18]. TVS will be placed and programmed before each exercise session being used during the whole session. In this vein, it is important to note that a systematic review showed that TVS has been shown to be safe and tolerable in multiple populations [44] with minimal side effects such as slight itching and redness of the skin [42]. The device will be configured with the same parameters and intensity described above for sham TVS condition. However, the device will be located in the left earlobe [45] (see Figure 2). This area does not have vagal innervation, and more importantly, it has been proven that it does not produce any activation in the cortex or brain stem [46].

*Exercise program*: Both groups will carry out a tailoring exercise program in 12 sessions, of approximately one hour, distributed in 3 sessions per week for a month. The exercise program will be administered in person, keeping a ratio of 1 physiotherapist per 3 participants at the most. The objectives of the program will be to reduce pain and improve functional capacity. The program includes a warm-up stage (i.e., stationary bicycle), a central stage of strengthening and stretching exercises of the knee, hip and lumbar zone muscles, and finally, a stage of returning to calm [47]. Each exercise will have 5 levels of difficulty (from 1 = easy to 5 = difficult). All participants will start at level 3, increasing or decreasing in level depending on the pain they experience and their ability to perform the exercises. In addition, the physiotherapist in charge of administering the programme will assess session by session whether the difficulty of the various exercises should be adjusted. (i.e., tailoring). This program was designed based on the findings of literature reviews and meta-analyses (Appendix A) [12,48,49,50].

Both groups will carry out the same exercise program. The physiotherapists will record the attendance to the treatment sessions and each patient’s progress in a record book.

If a patient does not attend on the stipulated date, he/she will be contacted by phone on the same day to find out the reason for the absence, and he/she will be gently invited to attend the next day to comply with the scheduled session. If a patient does not attend two consecutive sessions, the principal investigator will contact him/her to consult the reason for non-attendance and invite him/her to continue with the study. Regular face-to-face meetings will be held between one of the investigators and the treating physiotherapists to reinforce the intervention protocols and discuss study issues, which will help ensure the uniform application of the treatment.

In every session, the physiotherapists will make a brief evaluation to determine the progress of the exercise program. Additionally, the session could be rescheduled if pain or inflammation is preventing the correct performance of the session. In this case, the idea is to maintain 3 sessions per week at least. Adverse events that the patient attributes to the use of TVS or the exercise program will be analysed jointly by the treating physiotherapists and the patient to assess the possible discontinuation of the intervention.

### 3.6. Data Analysis

All statistical analyses will be performed using the statistical software program STATA v16. A descriptive analysis of the main variables of the study will be carried out using central tendency statistics (mean, median, mode, etc.) and dispersion statistics (standard deviation, variance, range, etc.). The baseline variables will be reviewed to evaluate the comparability of the groups. The primary outcome measure was the average pain VAS measured at 4 weeks after randomization. TVS was compared with TVS sham using an analysis of covariance (ANCOVA) model that included a TVS sham group as the factor of interest and the corresponding baseline average pain VAS as a covariate. The primary analyses included all available data from all randomized participants. Missing data were accommodated using the technique of multiple imputations. Statistical analyses will be performed according to the intention to treat principle. An alpha level of 0.05 will be accepted as significant. Results will be reported as a minimum between-group mean squared differences and 95% confidence intervals from baseline to end of follow-up.

A secondary analysis compared the percentage of “responders” between groups [51] using a chi-square test. A “responder” was defined as a participant whose VAS pain scores at 4 weeks improvement by at least 20% relative or 15 mm absolute from baseline or was below 40 of 100 [52]. Participants who prematurely discontinued were defined as non responders. Prespecified analyses of secondary outcome variables used similar ANCOVA models to those of the primary analyses. No adjustment for multiplicity was made in the secondary analyses, as they were considered exploratory and hypothesis generating.

### 3.7. Role of the Funding Source

The funders played no role in the design, conduct, or reporting of this study.

## 4. Discussion

KOA clinically presents with various symptoms, including pain, stiffness, instability, and significant limitations in daily activities [53]. This results in an explicit restriction in social participation and a subsequent decrease in quality of life. In this scenario, KOA treatment strategies range from pharmacological and physiotherapy treatments to surgical interventions [34]. However, in terms of their efficacy, they still show limitations.

In this context, it is essential to find new treatments for KOA. TVS has been proven as a suitable technique to inhibit pain [16,21]. Thus, this trial will generate evidence regarding the efficacy of TVS in pain perception in individuals with KOA. Finally, it should be noted that one limitation is that the therapists are not blinded to the patient’s treatment since they are in charge of placing the electrode in the ear, thus knowing in which group the patients are assigned. However, we do not believe that this will affect the intervention.

## 5. Conclusions

If the efficacy of TVS is confirmed, a new therapeutic tool may be included in the rehabilitation of individuals with KOA. This information will be useful as an input in the clinical decision-making on the use of TVS in individuals with KOA.

## Figures and Tables

**Table 1:** Schedule of recruitment, interventions and evaluations.

			Study Period			
Activity	Recruitment and Assignment		Post-Assignment Stage	End of the Intervention	Follow-Up Stage	
**Moment**	**−t1**	**0**	**Month 1**		**Month 2**	**Month 4**
**RECRUITMENT**						
Screening	**X**					
Informed Consent	**X**					
Assignment		**X**				
**INTERVENTION**						
Experimental group			**X**			
Control group			**X**			
**EVALUATIONS**						
Descriptive data		**X**				
Primary variable		**X**		**X**	**X**	**X**
Secondary variables		**X**		**X**	**X**	**X**
Other variables		**X**				

**Table 2:** Eligibility criteria.

Inclusion Criteria	Exclusion Criteria
Women and men aged 55 and over The medical diagnosis of knee osteoarthritis according to the clinical criteria of the American College of Rheumatologists Chronic pain (i.e., 3 months or more) Average pain of the last seven days among 30 mm and 80 mm on the Visual Analogue Scale Walking without technical aids or knee brace Availability to attend an exercise program three times a week for four weeks Access to communication via telephone Agreement to participate in the study by signing an informed consent form	Presence of the following symptoms and signs: Fever or chills, large effusions, paresthesias or paresis of the lower limb Presence of medically diagnosed psychiatric or neurological diseases (cognitive impairment, Alzheimer’s, Parkinson’s, epilepsy, moderate or severe depression, bipolar disorders, obsessive compulsive disorder, etc.) Neuromuscular or cardiac disorders (i.e., arrhythmia, conduction cardiac block), stroke, generalised rheumatology, diabetes mellitus II, morbid obesity (i.e., equal to or greater than III) Medical contraindication of physical exercise Joint infiltration or lower limb surgery in the previous 6 months Surgery is planned for the next 6 months Take strong regular two or more kinds of medication Conditions that affect the lower limb and/or limit its functional capacity, such as previous fractures in the area, deformity, joint replacement, tibial osteotomy Participation in exercise programs in the last nine months Inability to follow instructions

## Data Availability

Not applicable.

## Appendix A. Exercise Protocol

The exercise protocol is guided by general principles described below: 1.- The exercises should not increase the patient’s pain significantly. They can cause fatigue and a feeling of tiredness, but they should not increase the symptoms significantly; that is, if the patient feels the same pain when doing the exercise as when resting, he/she can do it without pain or with mild pain (VAS 30 mm. approximately). 2.- If a patient can exercise at one level without increasing their basal pain, he/she move to the next level. If the next level causes increased pain, the level should be decreased. 3.- Emphasis should be placed on the quality of the exercises’ execution and not on the repetitions or series. If a patient performs the exercise inappropriately, an attempt should be made to correct it. Stop the exercise if the patient is not able to continue with quality repetitions, that is, without compensation and in a coordinated and fluid manner. The patient should not perform apnea in isometric exercises. All exercises have the transversal objective of reducing pain.					
Objective	Level 1	Level 2	Level 3	Level 4	Level 5
Objective: To strengthen the quadriceps	Patient in a seated position. Isometric contraction of the quadriceps with the extended leg. Hold for 10 s and perform 10 repetitions. Alternating both legs.	Patient in supine position. Lifting of the extended leg. Hold for 10 s and perform 10 repetitions. Alternating both legs.	Patient in supine position. Lifting of the extended leg. Hold for 10 s and perform 10 repetitions with 2 kg at the distal part of the leg. Alternating both legs.	Patient in seated position. Knee extension with 2 kg at the distal part of the leg. 10 repetitions × 3 series. Alternating both legs.	Patient in seated position. Knee extension with 4 kg at the distal part of the leg. 10 repetitions × 3 series. Alternating both legs.
Objective: To strengthen the hamstrings	Patient in prone position. Knee flexion with no weights. 10 repetitions × 3 series. Alternating both extremities.	Patient in prone position. Knee flexion with 1 kg at the distal part of the leg. 10 repetitions × 3 series. Alternating both extremities.	Patient in standing position. Knee flexion with 1 kg at the distal part of the leg. 10 repetitions × 3 series. Alternating both extremities.	Patient in standing position. Knee flexion with 2 kg at the distal part of the leg. 10 repetitions × 3 series. Alternating both extremities.	Patient in standing position. Knee flexion with 4 kg at the distal part of the leg. 20 reps × 3 sets. Alternating both extremities.
Objective: To strengthen the gluteus maximus	Patient in prone position. Isometric contraction of the gluteus maximus with posterior extended leg lift. Hold for 10 s and perform 10 repetitions. Alternating both extremities.	Double leg supine bridge. Hold for 5 s and perform 10 repetitions.	Double leg supine bridge. Hold for 10 s and perform 10 repetitions.	Single leg supine bridge. Hold for 10 s and perform 10 repetitions. Alternating both extremities.	Single leg supine bridge whit unstable surface. Hold for 10 s and perform 20 repetitions. Alternating both extremities.
Objective: To strengthen the gluteus medius	Patient in lateral decubitus position. External rotation of the hip, with hip and knee flexion (clamshell exercise). 10 repetitions × 3 series. Alternating both extremities.	Patient in lateral decubitus position. Isometric lateral extended leg lift. Hold for 3 to 5 s × 10 repetitions. Alternating both extremities.	Patient in lateral decubitus position. Isometric lateral extended leg lift. 10 s × 10 repetitions. Alternating both extremities.	Patient in lateral decubitus position. Isotonic lateral extended leg lift. 10 repetitions × 3 series. Alternating both extremities.	Patient in lateral decubitus position. Isotonic lateral extended leg lift with 1 kg at the distal part of the leg. 10 repetitions × 3 series. Alternating both extremities.
Objective: To strengthen the lumbo-abdominal area	Patient in 4 prone position. Lifting of leg. Hold for 3 to 5 s and perform 10 repetitions. Alternating both extremities.	Bird dog. Hold for 3 to 5 s and perform 10 repetitions. Alternating every diagonal.	Bird dog. Hold for 10 s and perform 10 repetitions. Alternating every diagonal.	Knee prone bridge. Hold for 5 to 10 s and perform 10 repetitions.	Prone bridge. Hold for 5 to 10 s and perform 10 repetitions.
Objective: Co-contraction at CCC	Double-leg squats with parallel support. 10 repetitions × 3 series.	Double-leg squats. 10 repetitions × 3 series.	Single-leg squats 10 repetitions × 3 series. Alternating both extremities.	Single-leg squats. 20 reps × 3 sets. Alternating both extremities.	Single-leg squats. 30 repetitions × 3 series. Alternating both extremities.
Objective: Increase or maintain aerobic capacity	Cyclorgometer 10 min. individual ‘tolerance	Cyclorgometer 10 min. 9–10 Borg scale	Cyclorgometer 10 min. 11–12 Borg scale	Cyclorgometer 10 min. 13–14 Borg scale	Cyclorgometer 10 min. 15–16 Borg scale
Objective: Increase knee flexo-extension range	Patient in supine position, hip flexion with extended knee and band assistance to stretch hamstring muscles (2 repetitions of 30 s). Patient in supine position, knees to chest with the assistance of his/her arms to stretch gluteal and lumbar muscles (2 repetitions of 30 s). Patient in prone position, knee flexion with band assistance for stretching the quadriceps muscle (2 repetitions of 30 s). It should be noted that all stretches should be up to the feeling of tension and with no increase in pain.				
